# Co-expression network analysis of the transcriptomes of rice roots exposed to various cadmium stresses reveals universal cadmium-responsive genes

**DOI:** 10.1186/s12870-017-1143-y

**Published:** 2017-11-07

**Authors:** Mingpu Tan, Dan Cheng, Yuening Yang, Guoqiang Zhang, Mengjie Qin, Jun Chen, Yahua Chen, Mingyi Jiang

**Affiliations:** 1 National Key Laboratory of Crop Genetics and Germplasm Enhancement, Nanjing Agricultural University, Nanjing, China; 2 College of Life Sciences, Nanjing Agricultural University, Nanjing, China

**Keywords:** Transcriptome, Co-expression network, WGCNA, Cadmium, Rice root

## Abstract

**Background:**

The migration of cadmium (Cd) from contaminated soil to rice is a cause for concern. However, the molecular mechanism underlying the response of rice roots to various Cd stresses remains to be clarified from the viewpoint of the co-expression network at a system-wide scale.

**Results:**

We employed a comparative RNAseq-based approach to identify early Cd-responsive differentially expressed genes (DEGs) in rice ‘Nipponbare’ seedling roots after 1 h of high-Cd treatment. A multiplicity of the identified 1772 DEGs were implicated in hormone signaling and transcriptional regulation, particularly NACs and WRKYs were all upregulated under Cd stress. All of the 6 Cd-upregulated ABC transporters were pleiotropic drug resistance proteins (PDRs), whereas all of the 6 ZRT/IRT-like proteins (ZIPs) were consistently downregulated by Cd treatment.

To further confirm our results of this early transcriptomic response to Cd exposure, we then conducted weighted gene co-expression network analysis (WGCNA) to re-analyze our RNAseq data in combination with other 11 previously published RNAseq datasets for rice roots exposed to diverse concentrations of Cd for extended treatment periods. This integrative approach identified 271 transcripts as universal Cd-regulated DEGs that are key components of the Cd treatment coupled co-expression module. A global view of the 164 transcripts with annotated functions in pathway networks revealed several Cd-upregulated key functional genes, including transporter ABCG36/OsPDR9, 12-oxo-phytodienoic acid reductases (OPRs) for JA synthesis, and ZIM domain proteins JAZs in JA signaling, as well as *OsWRKY10*, NAC, and ZFP transcription factors. More importantly, 104 of these, including *ABCG36/OsPDR9*, *OsNAC3*, as well as several orthologs in group metalloendoproteinase, plastocyanin-like domain containing proteins and pectin methylesterase inhibitor, may respond specifically to various Cd pressures, after subtracting the 60 general stress-responsive genes reported to be commonly upregulated following multiple stresses.

**Conclusion:**

An integrative approach was implemented to identify DEGs and co-expression network modules in response to various Cd pressures, and 104 of the 164 annotatable universal Cd-responsive DEGs may specifically respond to various Cd pressures. These results provide insight into the universal molecular mechanisms beneath the Cd response in rice roots, and suggest many promising targets for improving the rice acclimation process against Cd toxicity.

**Electronic supplementary material:**

The online version of this article (10.1186/s12870-017-1143-y) contains supplementary material, which is available to authorized users.

## Background

Cadmium (Cd) is a nonessential detrimental element that poses potential chronic toxicity to living organisms [1–3]. Cd is absorbed by the roots from the soil and transported to the shoot, so understanding the molecular mechanisms of plant roots coping with challenges imposed by unfavorable Cd element is one of crucial steps for improving acclimation of plants against Cd toxicity [4].

Like other nonessential elements, Cd enters cells as hitchhiker through some transporters specific for nutrient elements [5]. For rice, natural resistance-associated macrophage protein 5 (OsNramp5) is the major transporter for Mn and Cd transport into the inner root [6, 7]. The iron-regulated transporter 1 (OsIRT1) and OsNramp1 are potentially involved in this process [8], but their contributions appear small compared to that of OsNramp5 [3]. Under the adverse condition imposed by heavy metals, plants have evolved correspondingly a multiplicity of highly integrated adjustments to reduce the toxicity of absorbed heavy metals [9, 10]. In plant roots, Arabidopsis heavy metal ATPase AtHMA2 and AtHMA4 [11], as well as the ATP-binding cassette (ABC) transporter AtPDR8 [12] and OsHMA2 [13] localize to the plasma membrane and transport Cd out of the cell [14]. Another ABC-type transporter AtABCC3 [15], AtHMA3 [16] and OsHMA3 [17–19] play important roles in Cd detoxification by sequestering Cd to root vacuoles [3, 14].

Besides the strategies of heavy metal sequestration and detoxification, various signaling pathways are stimulated by Cd stress, and plant transcription factors (TFs) play vital roles in the regulation of signal transduction and protection of cells from stresses [20].

Although microarray-based transcriptomic analysis did provide highly valuable information on the plant responses to Cd pressure over the past decade [21], the complex Cd defense system in plants was still unexplored. Currently, ever-increasing RNAseq-based comparative transcriptomic studies have been conducted in several plant species, such as radish [22], Cd/zinc/Pb co-hyperaccumulating *Crassulaceae* [23], fast growing Cd-resistant tree [24], and maize [25, 26]. Taking the staple model crop rice into account, a few transcriptomic studies focusing on genes involved in the response to Cd stress have been conducted with the aid of RNAseq approach [1, 20, 27, 28]. However, there is still lack of an integrative investigation on these dispersed transcriptomes of rice roots under various Cd pressures over diverse treatment periods.

In the current omics era, co-expression networks and gene regulatory networks are widely used to predict functional roles of individual genes at a system-wide scale [29]. WGCNA is such a fascinating genome-wide approach focusing on elucidating biological networks instead of individual genes [29–31].

This powerful approach has been widely used on a range of systems for elucidating various processes, including dissecting flower development in Arabidopsis [32, 33] and strawberry [30], characterizing wheat endosperm development [34] and soybean domestication [35], revealing biotic stress responses in Arabidopsis [36], citrus [37] and *Medicago truncatula* [38], elucidating the holistic picture of drought response in grapevine [39], Cd response in maize [26] and the hyperaccumulating ecotype of *Sedum alfredii* [23]. Interestingly, WGCNA was also utilized to confirm the genome-wide association study (GWAS) inferred candidate genes associated with NaCl tolerance in Arabidopsis [40].

Through integrating transcriptome and metabolite data, gene co-expression networks related to inositol phosphate in maize [41] and fruit anthocyanin content in apple [42, 43] were constructed by WGCNA. Recently, three types of omics data including 31,447 mRNAs, 13,175 proteins and 4267 phosphoproteins were grouped individually by WGCNA into co-expression modules, and then integrated into omics networks in a developmental atlas of maize [29]. In addition to these integrations of different levels of omics data of the same sample, expression datasets generated by different groups under different experimental conditions and biological systems can be integrated by WGCNA to aid the annotation of rice genome [44]. It was also implemented to elucidate the genetic basis of drought tolerance in rice [45, 46] and discover the core biotic stress responsive genes in Arabidopsis [47]. However, there is no such a comprehensive resource aiming to elucidate the scaffolding mechanisms for Cd signaling pathways in rice from the viewpoint of co-expression network at a system-wide scale.

In this study, we implemented a comparative RNAseq-based approach to identify Cd-responsive DEGs in rice seedling roots under 1 h of high-Cd stress. Among the 1772 DEGs responsive to short-term high-Cd, 536 are novel Cd-responsive genes. To further clarify our findings of the early transcriptomic response to Cd exposure, we utilized WGCNA to re-analyze our RNAseq data in combination with other 11 published RNAseq datasets with different Cd concentrations and different time points. A universal Cd-responsive DEGs subset of 164 annotatable genes was extracted from the co-expression network modules generated by WGCNA, and 104 of them were Cd-specific regulated genes which were not reported to be general stress-responsive previously. Our results provide insight into the high-confidence universal molecular mechanisms beneath the Cd response in rice roots, and the universal Cd-regulated DEGs that may contribute to plant responses to Cd stress will be valuable for further improving the Cd response in rice using the genetic engineering approach.

## Results

### Global transcriptomic changes of rice roots in response to short-term high-Cd stress

**Fig. 1:**
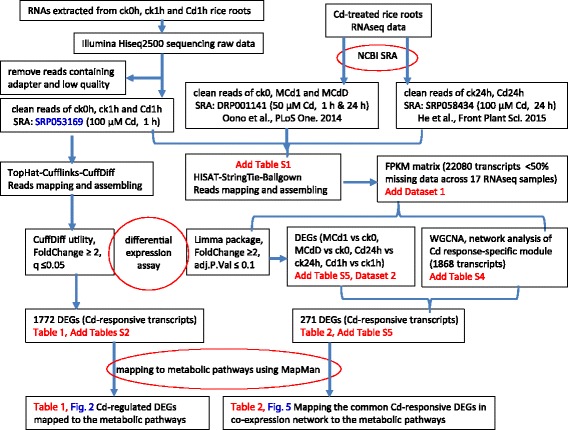
A flowchart showing the comprehensive RNAseq data analysis procedure

Transcriptomic changes in roots response to 1 h of high-Cd stress were determined by comparing the control (ck1h) and 100 μM Cd-treated (Cd1h) rice seedlings (Additional file 1: Table S1). After using the stringent criteria (fold change ≥ 2 and q_value ≤ 0.05) to select transcripts, a total of 1772 gene isoforms were identified as being early Cd-responsive (Fig. 1). Since few genes had more than one Cd-regulated transcript isoform, differentially expressed transcripts were regarded as differentially expressed genes (DEGs) (Table 1, Additional file 2: Table S2).

**Table 1:** Cd-regulated differentially expressed genes (DEGs) mapped to the metabolic pathways

BinName	FPKM		Cd1h/ck1h		Rice gene annotation
Transcript ID	ck1h	Cd1h	log2FC	q_value	
Jasmonate synthesis-degradation and signaling					
Os06g11290.1^△^	5.18	435.09	6.39	0.0019	12-oxophytodienoate reductase, ***OsOPR1***
Os06g11210.1^#^	58.99	717.03	3.60	0.0019	*OsOPR5* [Q69TI0]
Os06g11240.1	14.15	339.14	4.58	0.0019	OPR
Os06g11200.1	0.15	2.40	3.98	0.0188	OPR
Os03g55800.1^∑^	29.03	146.45	2.33	0.0019	allene oxide synthase, *AOS1*
Os03g12500.1^∑^	2.23	13.52	2.60	0.0019	allene oxide synthase, *AOS2*
Os12g05440.1*	1.85	58.66	***4.98***	0.002	cytochrome P450, ***CYP94C2b***
Os03g08310.1*	1.88	27.26	***3.86***	0.002	ZIM domain containing protein, ***OsJAZ9***
Os03g28940.1^△^	38.17	106.38	1.48	0.0019	ZIM domain containing protein
Os09g02710.1	0.63	4.20	2.73	0.0035	Arabidopsis loss of the timing of Et and JA biosynthesis 1
Abscisic acid synthesis-degradation and signaling					
Os03g44380.1^△^	0.53	5.75	3.43	0.0019	9-cis-epoxycarotenoid dioxygenase, ***OsNCED3***
Os07g05940.1^△^	0.59	99.97	7.41	0.0019	***OsNCED4***
Os12g42280.1^△^	2.63	32.42	3.62	0.0019	*OsNCED5*
Os12g29400.1^△^	5.88	14.09	1.26	0.0019	ABA-responsive protein [Q2QQY9]
Os04g44510.1	51.06	244.45	2.26	0.0019	ABA-responsive protein (GRAM domain)
Os04g44500.1	17.60	49.88	1.50	0.0019	ABA-responsive protein (GRAM domain)
Os10g04860.1	5.05	2.26	−1.16	0.0019	aldehyde oxidase
Os02g52780.2	0.71	14.01	4.30	0.0019	***OsbZIP23***
Ethylene signaling					
Os07g48630.1^△^	52.93	109.98	1.06	0.002	ethylene-insensitive 3
Os02g43790.1^△^	41.57	265.23	2.67	0.002	ethylene-responsive transcription factor, *OsBIERF3*
Os03g09170.1^△^	20.44	218.22	3.42	0.002	ERF
Os04g46220.1^Ⓜ^	44.26	119.27	1.43	0.002	ERF
Os08g31580.1	102.51	268.56	1.39	0.002	ERF
Os01g54890.1	4.75	31.18	2.71	0.034	ERF2
Os01g58420.1	49.28	134.22	1.45	0.002	***OsERF3***
Os11g06770.2*	0.10	0.70	***2.75***	0.027	*ERF110*
Os02g43970.1*	2.93	7.61	***1.37***	0.002	ABA-responsive DREB gene, ***ARAG1***
Os10g41330.2*	5.79	64.06	***3.47***	0.002	AP2 domain
Os06g10780.1*	2.17	6.55	***1.59***	0.002	AP2 domain
Os02g43940.1*	1.74	6.56	***1.92***	0.002	AP2 domain, *OsDREB4–1*
Os09g28440.1	0.59	47.37	6.32	0.002	AP2 domain, ***OsEATB***
Os08g36920.1^#^	0.76	70.32	6.53	0.002	AP2 domain
Os03g08460.1^Ⓜ^	91.75	42.47	−1.11	0.002	AP2 domain
Os03g08490.1	215.27	81.73	−1.40	0.002	AP2 domain
Os03g08500.1	538.17	256.44	−1.07	0.002	AP2 domain
Os04g08740.3	68.92	31.96	−1.11	0.002	ethylene receptor, *OsETR2* [Q7XX84]
Gibberellin synthesis-degradation					
Os06g37300.2	0.22	0.01	−2.36	1.1290	ent-kaurene oxidase 4, *OsKO4* [Q0DBF4]
Os03g63970.1*	18.68	0.00	***−1.12***	40.5099	gibberellin 20 oxidase 1
Os05g48700.1	2.84	0.01	1.34	1.1252	gibberellin 2-beta-dioxygenase
Metal binding, chelation and storage					
Os04g57200.1^△^	54.50	169.43	1.64	0.002	heavy metal transport/detoxification protein
Os04g57200.2	3.10	47.85	3.95	0.002	
Os04g17100.1^△^	2.63	32.28	3.62	0.0063	heavy metal-associated domain protein, HMAD HMAD
Os04g17100.2	43.72	123.38	1.50	0.0019	
Os03g05750.1^△^	0.54	89.45	7.38	0.003	HMAD
Os03g05750.2	9.01	212.68	4.56	0.002	
Os02g37290.1^△^	38.65	132.69	1.78	0.002	HMAD
Os02g37300.1^△^	11.63	66.20	2.51	0.002	HMAD
Os02g37330.1^△^	3.99	98.49	4.63	0.002	HMAD
Os04g39350.1^△^	4.57	33.87	2.89	0.002	HMAD
Os04g39010.1	0.94	3.69	1.97	0.022	HMAD
Os02g37320.1	2.57	19.20	2.90	0.002	HMAD
Os02g37320.2	9.45	84.16	3.15	0.002	
Os01g48710.1	116.94	46.15	−1.34	0.002	HMAD
Os03g64340.1*	5.31	12.44	***1.23***	0.0050	HMAD
Divalent cations transporters					
Os08g10630.1^△^	19.33	6.55	−1.56	0.0019	ZRT/IRT-like protein, *OsZIP4* [Q6ZJ91]
Os05g10940.1^Ⓜ^	40.47	19.81	−1.03	0.0019	*OsZIP7* [Q6L8F7]
Os05g39560.1	5.37	1.36	−1.98	0.0113	*OsZIP5* [Q6L8G0]
Os05g39560.2	40.35	9.21	−2.13	0.002	
Os06g37010.1	5.56	2.13	−1.39	0.0050	*OsZIP10* [Q5Z653]
Os05g39540.2*	97.22	25.45	***−1.93***	0.0019	*OsZIP9* [Q0DHE3]
Os03g46470.1*	14.68	2.46	***−2.58***	0.0076	Iron-regulated transporter 1, ***OsIRT1***
ABC transporters and pleiotropic drug resistance proteins					
Os01g42380.1^#^	1.35	5.99	2.15	0.0019	*ABCG36*/***OsPDR9*** *,* pleiotropic drug resistance protein
Os01g42410.1^∑^	3.66	21.96	2.59	0.0050	*ABCG37*/*OsPDR8* [Q8GU89]
Os02g11760.1^△^	0.34	2.90	3.10	0.0019	*ABCG39*/*OsPDR7* [Q8GU88]
Os01g42370.1	0.28	0.91	1.68	0.0019	*ABCG35*/*OsPDR11* [Q8GU92]
Os08g29570.1	0.32	8.42	4.72	0.0101	*ABCG44*/*OsPDR17* [Q6YW62]
Os08g29570.2	4.95	18.57	1.91	0.002	
Os01g61940.1*	10.43	25.90	***1.31***	0.0019	ABC transporter family protein
MATE family of citrate/H^+^ antiport transporters					
Os08g43654.1*	1.05	2.52	***1.26***	0.026	MATE efflux family protein
Os12g03260.1*	1.50	4.33	***1.53***	0.047	MATE
Os04g48290.1	3.72	8.19	1.14	0.002	MATE
Os02g45380.1	1.23	8.37	2.76	0.002	MATE
other transporters					
Os12g25200.1^Ⓜ^	123.93	50.25	−1.30	0.002	chloride transporter
Os02g36414.1	162.32	70.28	−1.21	0.002	transporter family protein
Os04g37970.1	118.36	43.01	−1.46	0.002	transporter family protein
Os07g24230.1	1.26	0.38	−1.71	0.014	integral membrane transporter family protein
Os01g14520.1	131.86	45.76	−1.53	0.002	dicarboxylate /malic acid transport protein
Os09g31130.1	1.58	7.13	2.17	0.035	citrate transporter
Os10g30790.1^△^	26.30	94.24	1.84	0.046	inorganic phosphate transporter, ***OsPT8***
Os10g30790.3*	25.00	80.55	1.69	0.016	
Mitochondrial electron transport					
Os07g37730.1^△^	0.15	9.07	5.96	0.0035	NADH-ubiquinone oxidoreductase
Os04g51150.1^△^	14.41	35.47	1.30	0.0019	*AOX1A*, alternative oxidase
Os04g51160.1^Ⓜ^	0.66	46.99	6.15	0.0019	*AOX1B*
Os10g39870.1	10.79	29.33	1.44	0.0019	Arabidopsis Transmembrane protein G1P-related 1
Os05g09550.1	1.37	4.81	1.81	0.0482	Der1-like family domain containing protein
Os02g37000.1*	6.37	16.29	***1.35***	0.0019	mitochondrial prohibitin complex protein 1
Heat-shock transcription factor (HSF) family					
Os09g35790.1*	0.76	6.99	***3.20***	0.0168	*OsHsfB2c* [Q652B0]
Os09g35790.2*	9.03	26.10	***1.53***	0.0019	
Os02g32590.1^△^	1.21	7.07	2.55	0.0019	*OsHsfA3* [Q6H6Q7]
Os02g32590.2	0.38	2.76	2.88	0.0218	
Os04g48030.1^△^	3.05	14.05	2.20	0.0178	*OsHsfB2a* [Q7XRX3]
Os10g28340.1	0.62	12.76	4.36	0.0019	***OsHsfA2c***
Os10g28340.2	3.72	15.96	2.10	0.0135	
Os03g12370.3	0.40	4.95	3.65	0.0101	*OsHsfA9* [Q10PR4]
Os08g43334.2	2.45	44.04	4.17	0.0019	*OsHsfB2b* [Q6Z9C8]
Os02g13800.1	15.01	50.05	1.74	0.0019	*OsHsfC2a* [Q6EUG4]
NAC domain transcription factor family					
Os01g50360.1*	2.52	1.09	***−1.21***	0.026	NAC domain containing protein
Os11g03300.2	16.07	38.93	1.28	0.002	***OsNAC10***
Os01g60020.1^∑^	5.77	25.25	2.13	0.002	***OsNAC4***
Os07g12340.1^Ⓜ^	12.81	455.79	5.15	0.002	*OsNAC3* [Q7EZT1]
Os03g60080.1^#^	88.35	942.97	3.42	0.002	***SNAC1***
WRKY domain transcription factor family					
Os02g08440.1^#^	2.03	125.38	5.95	0.0019	*WRKY71*
Os02g08440.4	24.26	179.27	2.89	0.0019	
Os05g27730.1^△^	101.45	430.59	2.09	0.0019	***OsWRKY53***
Os01g61080.1^△^	6.51	92.36	3.83	0.0019	***OsWRKY24***
Os01g09100.1^△^	29.32	195.59	2.74	0.0019	*OsWRKY10*
Os05g50610.2	0.89	2.44	1.45	0.0317	*WRKY8*
Os08g13840.2	130.82	404.49	1.63	0.0019	*WRKY25*
Os05g09020.1	1.47	13.65	3.21	0.0019	*WRKY67*
Os05g09020.2	9.62	51.47	2.42	0.0019	
Os11g29870.1	5.02	15.26	1.60	0.0019	*WRKY72*
Os09g25060.1	3.11	24.14	2.95	0.0019	***OsWRKY76***
Os01g60600.1*	0.42	1.68	***1.99***	0.0323	*WRKY108*

The Cuffdiff utility was used to evaluate the transcript differential expression between ck1h and Cd1h samples. The DEGs uniquely identified in our RNAseq samples are marked with stars. Rice genes with experimentally verified functions are presented in bold fonts. Rice gene annotation was also referred from UniProt (square brackets contain the accession number). The DEGs marked with pound signs were also upregulated by medium and low Cd stress in rice seedling roots in 3 previous reports [21, 27, 48]. The DEGs marked with sigma were also upregulated uniformly by the lowest Cd treatment [48] as well as in one of the two high-throughput transcriptome study [21, 27]. Those marked with triangle were regulated uniformly by Cd treatment in the two high-throughput studies, and those labeled M were detected only in the microarray analysis [21]

To validate the expression pattern of DEGs that resulted from RNAseq, 8 DEGs were randomly selected for qRT-PCR assay (Additional file 3: Table S3). As anticipated, the qRT-PCR results were basically consistent with those from RNAseq (Additional file 4: Fig. S1), suggesting that DEGs resulted from RNAseq were credible for further analysis.

For the 1334 Cd-induced and 438 Cd-repressed transcript isoforms in rice ‘Nipponbare’ roots, 643 and 84 of them were also reported to be up- and down-regulated by 1 h of 50 μM medium Cd treatment (MCd1), correspondingly [27], whereas 575 up- and 44 down-regulated isoforms were also DEGs with same regulated pattern in the microarray-based analysis of early transcriptomic responses to 25 μM Cd treatment (lowCd) for 3 h [21]. Taking multiple Cd stresses commonly regulated genes into account, 298 up- and 1 down-regulated DEGs also displayed the same regulatory pattern in the previous reports, both with MCd1 and lowCd treatments [21, 27] (Additional file 5: Figure S2, Additional file 6: Dataset 1).

Excluding the 1070 DEGs also identified in two previous reports, the remaining 702 DEGs (433 up- and 269 down-regulated) are uniquely identified in this study (Additional file 5: Figure S2). Although 166 have other isoforms differentially expressed in previous two studies, 536 DEGs regulated by short-term high-Cd treatment are novel Cd-responsive genes (Table 1, Additional file 2: Table S2).

### Functional characterization of early Cd-responsive DEGs in rice roots

To gain insights into the functionality of the 1772 DEGs that are likely to be associated with the Cd response, all of these Cd-responsive transcripts were visualized in the candidate pathway networks with MapMan.

**Fig. 2:**
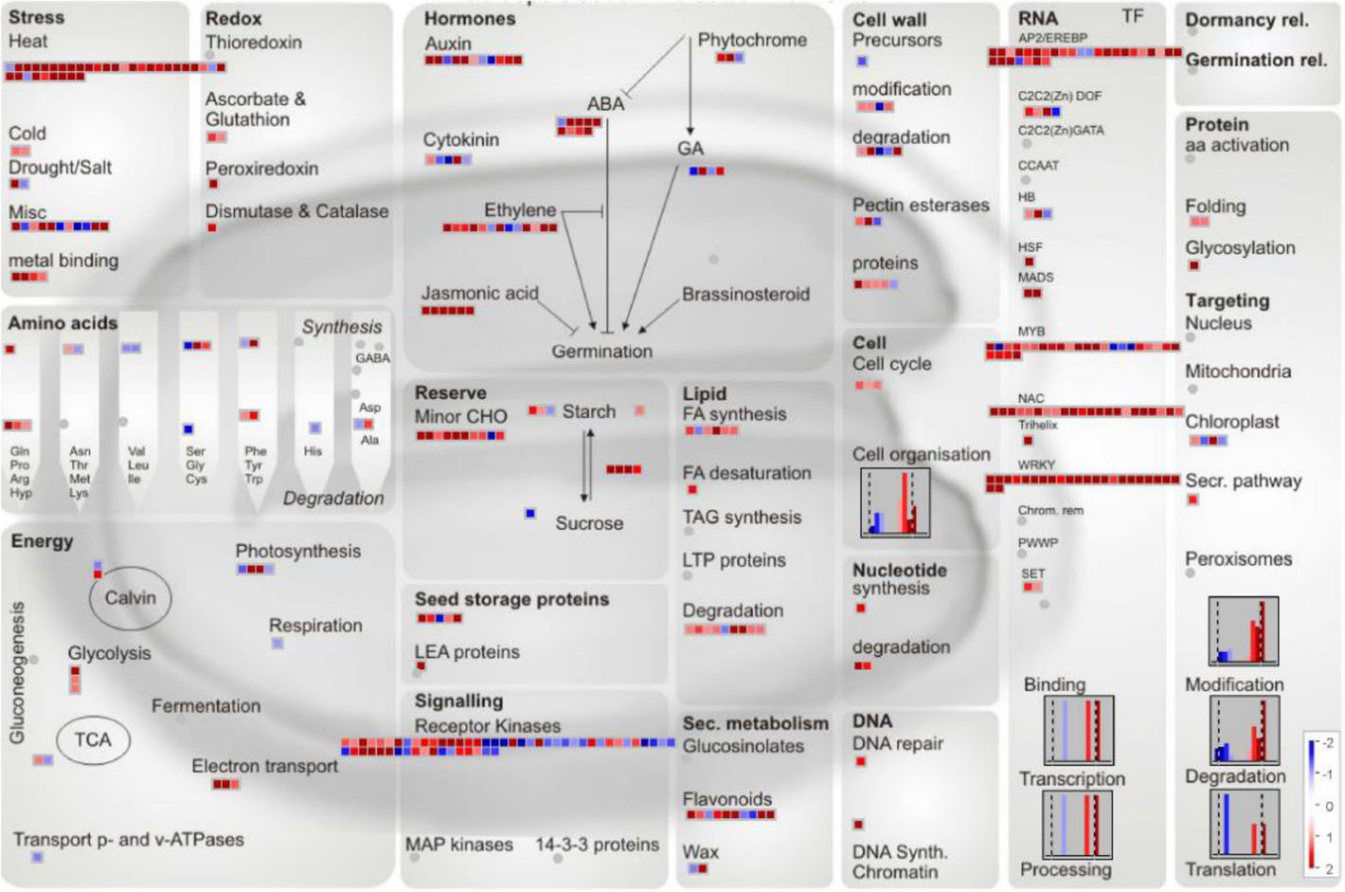
Global view of 1772 DEGs involved in diverse metabolic pathways in rice roots under Cd stress. Among the 1759 data points of 1772 DEGs, 603 data points were mapped on the metabolic pathways (Arabidopsis seed-Molecular Networks) using MapMan software. The colored boxes indicate the Log2 ratio of Cd1h/ck1h

Globally, the overrepresented biological functional pathway genes among Cd-regulated DEGs were involved in stress and hormone-signaling transduction, redox balance, regulation of transcription, ion transport, etc. (Fig. 2, Additional file 2: Table S2), which is consistent with previous studies [1, 21, 27]. Furthermore, the regulation patterns of JA signaling nodes, ABA-dependent pathway components, transporters, and TFs were essentially the same as those reported in previous Cd-treated rice transcriptomic studies (Fig. 2, Additional file 2: Table S2); therefore, these DEGs can be regarded as members of universal Cd-responsive genes.

Among the DEGs within the ‘Stress’ group, all transcripts involved in cold stress response and metal binding, and the majority of heat shock protein transcripts, were upregulated in response to Cd. Of the transcripts mapped to ‘Hormones’ category, 9 DEGs in Jasmonate signaling, 8 in ABA signal transduction, and 10 of 11 in Auxin signal transduction, as well as the majority of isoforms mapped to ethylene signaling, were upregulated by Cd treatment.

Additionally, 6 transcripts that encode antioxidant enzymes and thioredoxin, and the majority of TFs, including 7 members of the HSF family, 4 NACs, and 22 WRKYs, were all upregulated under Cd stress (Table 1, Fig. 2, Additional file 2: Table S2).

Based on the transport overview, 3 groups of mapped transporters (including 6 ABC transporters mainly in the PDR category, 6 amino acid transporters, and 3 ammonium transporters) were all upregulated quickly following Cd treatment. Ten of 11 heavy metal-associated domain containing proteins (HMADs) and 4 members of multidrug and toxic compound extrusion (MATE) efflux family were all rapidly upregulated in Cd-treated rice roots. However, all 6 zinc or iron-regulated transporters (ZRT/IRT-like proteins, ZIPs) including *OsIRT1* and *OsZIP4* were downregulated by Cd stress. Similarly, 5 other transporters, including 1 chloride transporter and 1 malic acid transport protein were also inhibited by short-term high-Cd stress (Table 1, Additional file 7: Figure S3).

With regard to the novel Cd-responsive DEGs, *CYP94C2b* and *OsJAZ9* were involved in Jasmonate signaling, one ethylene-responsive transcription factor (*ERF110*) and other 4 DEGs were involved in ethylene signaling, and the Cd-inhibited gibberellin-20 oxidase was responsible for gibberellin synthesis. Seven transporters are also putative novel Cd-responsive DEGs: these include 1 HMAD, *OsZIP9*, and *OsIRT1*, 2 MATEs, and 1 inorganic phosphate transporter OsPT8. Three TFs, including *OsHsfB2c*, 1 NAC, and *WRKY108*, are also among the novel Cd-responsive DEGs (Table 1, Additional file 2: Table S2).

Five DEGs, including one 12-oxophytodienoate reductase (*OsOPR5*), *ABCG36/OsPDR9*, stress responsive NAC (SNAC1) and *WRKY71* transcription factors genes, were also reported to be upregulated by medium and low Cd stresses in rice seedling roots in 3 previous reports [21, 27, 48]. In the latter 2 previous high-throughput transcriptome studies, 30 DEGs were upregulated whereas *OsZIP4* was downregulated by Cd treatment. Interestingly, OsNAC4 was also consistently upregulated in microarray analyses with 2 lowest Cd treatments, both at the 3-h time point. Transcription factors *OsbZIP23* and *OsNAC10* have also been previously shown to be responsive to medium Cd stress in RNAseq analysis [27], but in different gene isoforms, and mainly as the first isoforms (Table 1, Additional file 2: Table S2).

### Identification of DEGs and co-expression network modules in response to various Cd pressures

To investigate whether the early Cd-responsive genes identified in this study are involved in response to diverse concentrations of Cd treatments at different time points or not, 17 RNAseq samples covering those published and our RNAseq data of Cd-treated rice seedling roots (Additional file 1: Table S1) were processed by the new pipeline HISAT-StringTie-Ballgown. After filtering those transcripts with no more than 50% missing data, 22,080 transcripts across 17 RNAseq samples (Additional file 8: Dataset 2) were selected for analysis of DEGs and construction of gene co-expression network simultaneously.

**Fig. 3:**
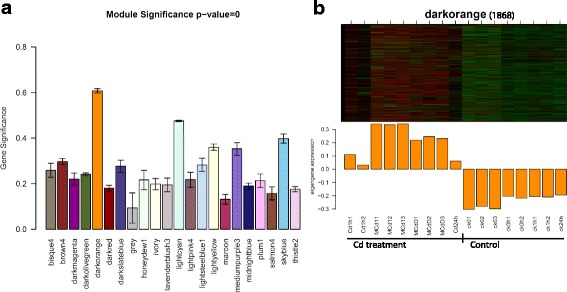
Gene co-expression network analysis of 17 RNAseq datasets for rice roots exposed to diverse concentrations of Cd for extended treatment periods. **a** Barplot of module significance. **b** Eigengene expression profile for the Cd-coupled module in 17 RNAseq samples. The module significance is defined as the mean gene significance across all genes in the module. For the heatmap of the expression of eigengenes in the Cd-coupled module (rows) across 17 RNAseq samples (see Methods section), low values means a lot of module genes are under-expressed (green color in the heatmap) and high values for over-expressed (red in the heatmap) in this samples. The number of genes in the module is indicated in parenthesis

For differential expression analysis, the stringent criteria (fold-change ≥ 2 and adj.P.Val ≤ 0.05) was used to select transcripts, thus 4 datasets containing DEGs [(1 h of Medium Cd treatment) MCd1 vs ck0, (1 d of Medium Cd) MCdD vs ck0, Cd24h vs ck24h and Cd1h vs ck1h)], representing various concentrations of Cd treatments across diverse time points, were obtained for further analysis (Additional file 9: Dataset 3). Meanwhile, the WGCNA package was employed to construct gene co-expression network of Cd response from the expression matrix of 22,080 transcripts across 17 RNAseq samples. This approach resulted in 22 distinct co-expressed modules (labeled by different colors), and the module ‘darkorange’ (with 1868 transcripts) is of particular interest (Fig. 3, Additional file 10: Table S4). This module has the highest level of module significance, and its module Eigengene expression profile suggests that it is correlated with Cd treatment, therefore, it is considered as the dominant Cd response-specific or Cd-coupled module.

**Fig. 4:**
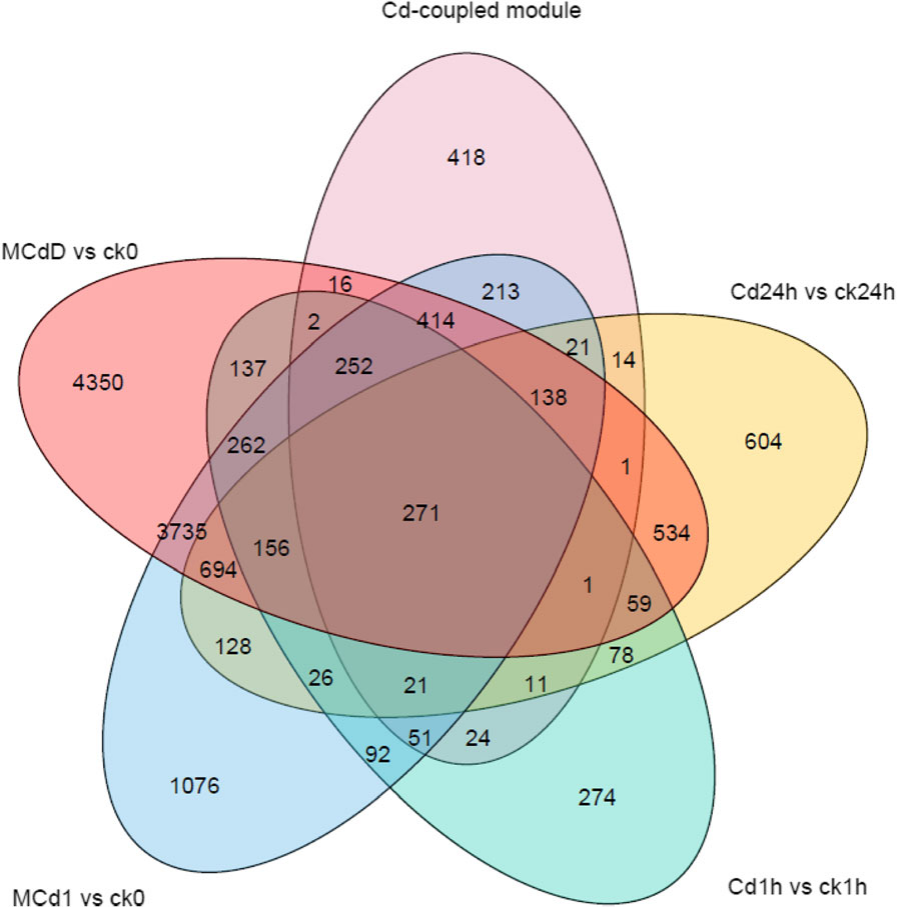
A Venn diagram showing the intersections of transcripts in the Cd coupled module and DEGs in different concentrations of Cd treatments across diverse time points. Rice roots samples from diverse Cd treatments and their corresponding control were compared in couples to analyze DEGs using the limma software package

The comparison of transcriptomes across different time points and various dosages of Cd treatments might provide additional information about gene function, therefore the intersection of transcripts in Cd-coupled module and the 4 DEGs datasets was selected for functional analysis (Fig. 4).

### Functional characterization of universal Cd-responsive DEGs in co-expression network

**Fig. 5:**
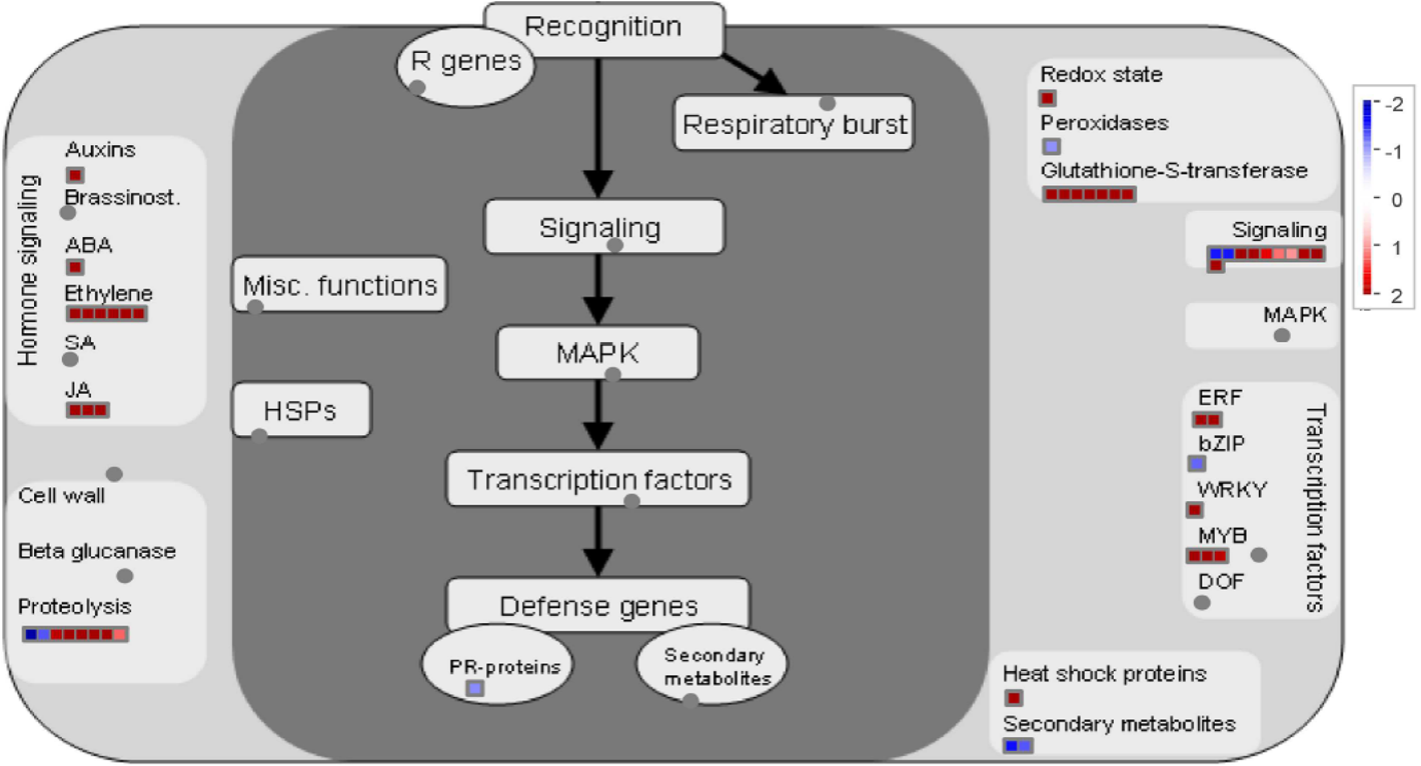
Global view of the 164 universal Cd-responsive DEGs involved in diverse metabolic pathways in rice roots under Cd stress. Among the 169 data points of 164 universal Cd-responsive DEGs, 51 data points were mapped on the metabolic pathways (Biotic stress) using MapMan software. The colored boxes indicate the Log2 ratio of Cd1h/ck1h

After the comparison with the 4 DEGs datasets, 418 of the 1868 transcripts from Cd-coupled module were unveiled to be unrelated to Cd response, whereas 271 transcripts were universal Cd-responsive DEGs, constituting the central hub of Cd response-specific module subset (Fig. 4). The global function view showed that 164 of 271 DEGs from the Cd response-specific module across 17 samples can be annotated and located in MapMan pathway network (Fig. 5, Additional file 11: Table S5) and the remaining 107 unmapped were novel transcripts with “MSTRG” prefix (Additional file 10: Table S4) assigned by StringTie.

Taking the “Biotic stress” pathways as an example, all of the common upregulated transcripts were enriched in category ‘Hormone Signaling’, ‘proteolysis’, ‘Redox’, ‘Signaling’ and ‘transcription factors’. In detail, the expression of genes implicated in ‘Jasmonic acid’ and ‘Transcription factors’ (WRKYs and ERFs) were upregulated in Cd treated roots (Fig. 5). Among the 164 common data points, 6 of them involving in ethylene signaling, 8 in JA synthesis and signal transduction, 7 transcription factors, 7 zinc finger proteins (ZFPs), 7 glutathione-S-transferases (GSTs) and 4 transporters were all upregulated by Cd stress (Table 2).

**Table 2:** Mapping the universal Cd-responsive DEGs in co-expression network to the metabolic pathways

BinName	Log2Foldchange				Rice gene annotation
Transcript ID	Cd1h	MCd1	MCdD	Cd24h	
Stress protein					
Os03g16020.1^Ⓜ^	4.78	8.93	8.95	6.76	hsp20/alpha crystallin family protein
Abscisic acid synthesis					
Os07g05940.1^△Ⓒ^	5.63	8.61	8.60	7.32	9-cis-epoxycarotenoid dioxygenase, ***OsNCED4***
Jasmonate synthesis and signaling					
Os06g11290.1^△Ⓒ^	5.06	6.25	5.76	4.72	12-oxophytodienoate reductase, ***OsOPR1***
Os06g11240.1	4.52	5.13	4.38	4.79	OPR
Os06g11210.1^#^	3.55	5.19	4.91	3.34	*OsOPR5*
Os10g25290.1^#Ⓒ^	2.72	5.54	4.66	2.97	ZIM domain containing protein, OsJAZ12
Os03g08330.1^△Ⓒ^	3.36	7.21	6.93	2.02	*OsJAZ10*
Os10g25230.1^△Ⓒ^	4.89	7.43	7.38	4.45	*OsJAZ13*
Os03g08310.1*	2.99	5.56	4.67	1.86	***OsJAZ9***
Os12g05440.1*^Ⓒ^	4.09	6.83	5.28	4.33	cytochrome P450, ***CYP94C2b***
Ethylene signal transduction					
Os08g36920.1^#^	5.16	8.29	8.90	5.47	AP2 domain containing protein
Os07g22730.1^Ⓜ^	3.17	5.98	7.58	3.40	AP2 domain
Os09g39850.1^Ⓜ^	3.71	5.30	5.72	2.72	AP2 domain
Os09g28440.1 ^Ⓒ^	4.67	7.54	7.97	6.41	AP2 domain, ***OsEATB***
Os02g43790.1^△Ⓒ^	2.57	3.89	4.36	2.09	ethylene-responsive transcription factor, *OsBIERF3*
Os05g34730.1	4.99	7.43	8.95	4.88	ethylene-responsive transcription factor, ***SERF1***
Transcription factor family					
Os01g09100.1^△^	2.44	4.65	4.68	3.14	OsWRKY10
Os01g01870.1^△^	2.53	3.48	1.77	1.58	HLH DNA-binding domain containing protein
Os01g49160.1^△^	1.91	4.07	3.91	2.11	MYB family transcription factor
Os01g19330.1 ^Ⓒ^	4.33	7.48	7.53	5.03	MYB
Os02g41510.1^#Ⓒ^	2.96	4.31	3.82	3.01	MYB
Os03g60080.1^#Ⓒ^	3.67	5.08	5.16	3.48	***SNAC1***
Os07g12340.1^Ⓜ^	4.93	6.26	5.91	3.04	*OsNAC3*
Os09g31390.1	−1.25	−1.46	−2.64	−1.64	*OsbZIP74*
Zinc finger proteins					
Os09g21710.1^#Ⓒ^	2.71	6.17	6.95	2.94	AN1-like zinc finger domain containing protein
Os03g32230.1^#Ⓒ^	5.04	7.77	6.03	3.61	ZOS3–12 - C2H2 zinc finger protein
Os03g60560.1 ^Ⓒ^	7.11	9.63	11.52	5.82	ZOS3–21, ***ZFP182***
Os12g39400.1^△Ⓒ^	2.36	5.08	5.67	2.75	ZOS12–09, *ZFP252*
Os03g60570.1^△Ⓒ^	5.12	9.13	8.86	5.79	ZOS3–22
Os02g45780.1^△Ⓒ^	2.74	5.76	5.24	2.48	zinc finger, C3HC4 type
Os09g29310.1^△^	3.83	6.05	5.86	2.33	zinc finger, C3HC4 type
Glutathione S transferases					
Os10g22070.1*	2.42	3.04	3.92	2.07	glutathione S-transferase, GST
Os09g20220.1^#Ⓒ^	6.43	7.67	7.73	8.32	GST
Os10g38340.1^△Ⓒ^	6.40	11.52	13.14	7.08	GSTU6
Os10g38720.1^Ⓜ^	2.87	5.78	7.45	4.64	GST
Os03g57200.1	2.10	2.66	2.93	1.35	GST
Os10g38314.1	5.71	7.11	8.28	5.29	GST
Os10g38350.1	1.97	3.07	1.94	2.20	GST
ROS regulation-related genes					
Os07g08840.3	5.09	5.39	3.94	4.17	apoplastic H-type thioredoxin*,* ***OsTRXh1***
Os04g33390.1*	1.98	3.42	2.26	2.24	prephenate dehydratase / arogenate dehydratase
Os11g42989.1*	5.09	5.40	6.16	3.81	exo70 exocyst complex subunit
Transporters					
Os01g42380.1^#^	2.07	4.51	4.56	4.00	*ABCG36*/***OsPDR9***
Os02g37300.1^△^	2.33	4.56	7.04	3.61	HMAD
Os02g37330.1^△^	4.25	7.19	8.63	4.43	HMAD
Os03g11900.1^Ⓜ^	1.15	2.78	2.58	1.99	monosaccharide transporter, *OsMST4*
others DEGs with Cd-upregulated homologs					
Os02g50730.1^△^	1.90	2.67	3.49	4.41	APK_ORTHOMCL1642, metalloendoproteinase
Os06g13180.1^△^	3.03	1.88	5.44	5.03	APK_ORTHOMCL1642, metalloendoproteinase
Os08g04350.1^△^	4.22	4.28	7.54	7.52	APK_ORTHOMCL3089, plastocyanin-like
Os08g04360.1	4.76	4.90	7.71	8.40	APK_ORTHOMCL3089, plastocyanin-like
Os08g04370.1^△^	3.04	3.02	5.56	4.75	APK_ORTHOMCL3089, plastocyanin-like
Os12g18560.1^△^	2.53	3.31	4.95	4.96	APK_ORTHOMCL14694, pectin methylesterase inhibitor
others novel DEGs					
Os01g61990.1*	1.42	1.55	1.15	1.70	ankyrin repeat-containing protein, *OsNPR4*
Os03g08520.1*	2.79	4.38	5.32	2.02	DUF581 domain containing protein
Os03g41330.1*	−1.59	−2.77	−3.08	−1.97	DUF260 domain containing protein
Os02g25700.1*	−1.31	−2.17	−3.04	−1.23	O-acyltransferase
Os03g08720.1*	−1.57	−1.75	−3.10	−3.27	transferase family protein
Os04g43710.1*	−1.23	−2.12	−2.30	−2.63	CAMK, Ca/CaM-depedent protein kinases
Os06g11030.1*	2.13	4.94	4.84	2.38	EF hand family protein
Os08g42490.1*	3.47	4.65	7.06	3.91	DC1 domain containing protein
Os12g43870.1*	3.05	4.39	3.81	3.20	expressed protein

Rice seedling roots samples of medium Cd stress (50 μM) for 1 h and 24 h (designated MCd1 and MCdD, respectively), high Cd pressure (100 μM) for 24 h (labeled as Cd24h) and 1 h (Cd1h, prepared in our lab), and their corresponding control are compared in couples to analyze DEGs using the limma package. The DEGs marked with stars are the uniquely identified DEGs in our RNAseq samples (Cd1h). Those marked with pound signs were also upregulated by medium and low Cd stress in rice seedling roots in 3 previous reports [21, 27, 48], and those with triangle were regulated uniformly by Cd treatments in the two high-throughput studies [21, 27], whereas those labeled M were detected only in the microarray analysis [21]. Those labeled C were general stress-responsive genes that were commonly upregulated by multiple stresses as previously reported [27]. Rice genes with experimentally verified functions are presented in bold fonts, and the homologous DEGs can be categorized into orthologous groups are presented with APK_ORTHOMCL number [86]

Of particular interest is the identification of 1 key enzyme 9-cis-epoxycarotenoid dioxygenase (*OsNCED4*) for ABA synthesis, 2 key enzymes (*OsOPR1* and *OsOPR5*) for JA synthesis, 6 transcription Factors including *OsWRKY10* and *SNAC1*, 6 ZFPs, 3 GSTs and 3 transporters including *ABCG36/OsPDR9*, which were also uniformly upregulated by medium and low Cd stresses in rice seedling roots in at least 2 previous reports [21, 27, 48]. Additionally, one C2H2-type zinc finger protein (*ZFP182*) was significantly enhanced even at the lowest Cd concentration [48], and it was strongly induced from FPKM of 5 to 858 after the short-term high-Cd treatment (Table 2, Additional file 2: Table S2).

After subtracting the 60 general stress-responsive genes reported to be commonly upregulated following multiple stresses [27], 90 universal Cd-upregulated and 14 Cd-downregulated DEGs may respond specifically to various Cd pressures (Table 2; Additional file 11: Table S5). Regarding the 104 Cd-specific regulated genes, several DEGs could be categorized into orthologous groups of model plants (numbered as APK_ORTHOMCL). Among these DEGs, two metalloendoproteinases were in APK_ORTHOMCL1642, three plastocyanin-like domain containing proteins in APK_ORTHOMCL3089, while one pectin methylesterase inhibitor (Os12g18560, *OsPMEI46*) in APK_ORTHOMCL14694 (Table 2; Additional file 11: Table S5).

It is noteworthy that 14 of the 104 Cd-responsive members are also novel DEGs, only identified in this study through combing the two approaches. Among these 14 novel Cd-responsive DEGs, *Os04g33390* (encoding prephenate dehydratase) is the ortholog of Arabidopsis arogenate dehydratases (Table 2, Additional file 2: Table S2, Additional file 11: Table S5).

## Discussion

### Early high-Cd responsive transporters in rice roots

To elucidate the rice early response to high-Cd stress, we performed pair-end RNAseq of rice roots with replicates treated with high Cd concentrations (100 μM) for 1 h, in parallel with control samples at the same time point. As expected, 702 of the 1772 DEGs were uniquely identified in our rice roots treated by short-term high-Cd treatment (Additional file 5: Figure S2, Additional file 2: Table S2), which differed from previous transcriptomic studies using short-term and moderate or low Cd treatment [21, 27].

Metal transporters play key roles in the acquisition, distribution, and homeostasis of Cd in plants [5]. Several Arabidopsis ABC transporters are suggested to mediate vacuolar sequestration of Cd-phytochelatin conjugates [15, 49]. In this study, all of the 6 Cd-upregulated *ABCGs/PDRs* were from one of the most highly represented subfamilies of ABC transporters (Table 1, Additional file 7: Figure S3).

In Arabidopsis, ABC transporter of the mitochondria AtATM3 [50] and AtABCC1–3 [15, 49] transported Cd into the vacuoles to increase Cd tolerance. AtABCC3 confers Cd tolerance by transporting phytochelatin (PC)-Cd complexes into the vacuoles to detoxify Cd [15]. In rice, the Cd-inducible *OsABCG43* is likely to sequester Cd at the subcellular level [51], similar to vacuolar sequestration mediated by OsHMA3 [17].

Among the Cd-upregulated ABCG-type transporter genes, *ABCG36/OsPDR9* is reported induced rapidly and markedly in rice roots by Cd and Zn [52]. Taking its homologs in Arabidopsis into consideration, AtPDR12 and AtPDR8 are all involved in heavy metal resistance, and the Cd-inducible *AtPDR8* can export multiple functionally unrelated substrates, including auxinic compounds and Cd; thus, it confers heavy metal resistance as a Cd extrusion pump [12], strengthening the potential of employing *ABCG36/OsPDR9* for genetic improvement of Cd tolerance in rice. Besides its mechanism as a pump to exclude lead, AtPDR12 functions as a plasma-membrane ABA-uptake transporter [53].

In addition to the upregulated PDR-type transporters, 4 members of the MATE efflux family were rapidly upregulated in 1-h Cd-treated rice roots (Table 1).

MATE proteins play a role in aluminum tolerance by mediating citric acid efflux from root cells to chelate Al [54]. Functional MATE homologs associated with Al tolerance were identified in Arabidopsis [55], *Medicago truncatula* [56], sorghum [57], barley [58, 59], wheat [60], maize [61], and rice (*OsFRDL4*, Os01g69010) [62].

In Arabidopsis, the MATE-related transporter DETOXIFICATION 1 (AtDTX1) has been described as an efflux transporter that detoxifies plant-derived or exogenous toxic compounds including Cd from the cytoplasm [63]. Interestingly, a recent study on mammalian MATEs has provided the first evidence that MATE transporters are involved in the cellular transport and detoxification of Cd [64]. Therefore, it would be worth investigating the role of the Cd-induced plant MATE transporters in combating Cd toxicity *in planta*.

Ten of 11 heavy metal-associated domain containing proteins (HMADs) were rapidly upregulated in Cd-treated rice roots (Table 1).

HMADs, such as AtHMAD1 [65] and heavy metal-associated isoprenylated plant proteins (HIPPs) metallochaperones [66], have been suggested play roles in metal binding and/or transport, thus regulating various processes under (a) biotic stress [65]. The main hypothesis for the mechanism of Cd homeostasis is that HIPPs protect the plant by trapping free Cd ion (via the CXXC core motif), thus preventing the ion from binding to a more essential protein [66, 67]. Whether these 10 Cd -upregulated rice HMADs are involved in Cd homeostasis and detoxification is of particular interesting.

With regard to the Cd-downregulated divalent cations transporters (Table 1, Additional file 7: Figure S3), 4 ZRT/IRT-like protein genes (*OsZIP4*, *OsZIP5*, *OsZIP9*, and *OsZIP10*) were consistently upregulated in the overexpression *OsHMA3* line [19].

Overexpression of *OsHMA3* enhanced the tolerance to Cd toxicity by increasing vacuolar sequestration of Cd in the roots, meanwhile, OsHMA3 is also responsible for vacuolar sequestration of Zn, but the translocation of Zn to the shoots is maintained by up-regulating these ZIP genes implicated in Zn uptake and translocation in the *OsHMA3*-overexpressed line [19].

Cd absorption from the soil is thought to occur mainly via ZIP family transporters in Zn/Cd-hyperaccumulators [10]. Among these, IRT1 protein is a broad-range metal ion transporter in plants. Arabidopsis AtIRT1 has previously been shown to transport Fe, Mn, Zn, Co, and Cd, whereas rice OsIRT1 and its homolog in barley HvIRT1 is able to transport Fe, Zn, and Cd [68–70]. However, OsIRT1 was neglected in 2 previous Cd-treated root transcriptomic studies, and it was found here to be suppressed by Cd treatment (Table 1, Additional file 2: Table S2). Moreover, the over-expression of *OsIRT1* led to increased iron and zinc accumulation in rice, but Cd and other metal content in flag leaves and mature seeds from transgenic plants did not differ from those of WT plants, both growing in a paddy field not severely contaminated by Cd [69]. These findings again emphasize the potential use of these 4 Cd-suppressed ZIPs in Cd-contaminated soil to balance micronutrient fortification and toxicity reduction, which might be achieved by overexpressing them in rice.

### Universal Cd-responsive genes in rice roots

To determine the universal Cd-responsive genes in rice ‘Nipponbare’ roots, we simultaneously performed a DEGs assay and gene co-expression network analysis. This combined approach produced 271 DEGs, which were putative universal Cd-responsive genes, and 164 DEGs of these with annotation information could be mapped to the MapMan pathway network (Table 2, Fig. 5, Additional file 11: Table S5).

Twelve mappable universal Cd-responsive DEGs were upregulated uniformly in previously published 3 reports including 2 microarray analyses, with the 2 lowest Cd treatments at the 3-h time point (Table 2, Additional file 11: Table S5) [21, 48], which demonstrated their high sensitivity to a wide range of Cd stresses across diverse time points. These included *OsOPR5* and *OsJAZ12* in JA signaling, the intensively studied *SNAC1*, 2 *ZFPs*, and *ABCG36/OsPDR9*. It is noteworthy that 14 universal Cd-responsive members are also novel DEGs only identified in this study through combining the 2 approaches (Table 2, Additional file 2: Table S2), and these include 2 experimentally verified genes, *OsJAZ9* and *CYP94C2b*, involved in JA signaling [71–73].

From the perspective of global pathway distribution, 3 *OPRs* for JA biosynthesis and 4 *OsJAZs* for JA signaling were uniformly upregulated in all Cd-treated rice root samples (Fig. 5, Table 2). The universal Cd-responsive *OsOPR1* identified here was also rapidly upregulated in leaves by heavy metals, Cu, Cd, and mercury [74]. Additionally, 2 maize *zmOPRs* were significantly upregulated in 2 maize genotypes following Cd stress [26]. Interestingly, *CYP94C2b*, in which overexpression can alleviate the JA response through inactivating JA and enhancing salt tolerance in rice [71], is also a universal Cd-responsive gene. Besides 3 *OPRs* and *CYP94C2b* for bioactive JA control, 4 *OsJAZs* for JA signaling were also shown to be universal Cd-responsive genes. As was expected, these 4 OsJAZs were also induced significantly by medium-term of medium Cd treatment (60 μM for 6 h) [28]. Among these, the stress-inducible *OsJAZ9* acts as a transcriptional regulator in JA signaling and modulates salt stress tolerance in rice, and *OsJAZ9*-suppression rice plants exhibited increased sensitivity to JA, resulting in reduced salt tolerance [72, 73].

With regard to universal Cd-responsive TFs (Table 2, Fig. 5, Additional file 11: Table S5), *SNAC1*/*OsNAC19* can be more strongly induced by exogenous application of JA than by ABA and ethylene [75], and *OsWRKY10* can be strongly upregulated after JA treatment [76]. Collectively, the synchronized expression patterns of *OsOPRs*, *OsJAZs*, and JA-responsive TFs genes (*SNAC1* and *OsWRKY10*) clearly indicate that the JA signaling pathway is one of the crucial elements in the plant response to Cd stress [21, 48]. Further identification of the upstream and downstream relationships between JA biosynthesis, and these TFs may contribute to elucidating the role of the JA-mediated signaling pathways underlying the responses of plants to Cd stress.

Beside *SNAC1* and *OsWRKY10*, 7 *ZFPs* were also universally upregulated under 4 Cd exposure conditions (Table 2, Additional file 11: Table S5). Several ZFPs have been demonstrated to regulate plant tolerance to heavy metals. Among them, bean PvMTF-1 confers Cd tolerance in tobacco through activation of tryptophan biosynthesis, and the zinc finger-like motif is essential for the metal-responsive element binding of PvMTF-1 [77]. Maize zinc finger protein OXIDATIVE STRESS2 can enhance Cd tolerance in Arabidopsis [78], and Arabidopsis zinc-finger transcription factor ZAT6 positively regulates Cd tolerance through the glutathione-dependent pathway [79]. Arabidopsis STOP1 (sensitive to proton rhizotoxicity 1) operates in the signal transduction pathway controlling acid-soil tolerance [80]*.* Another zinc finger ART1 (for Al resistance transcription factor 1) regulates 31 genes implicated in Al tolerance in rice [81]. Regarding the 7 universal Cd-responsive *ZFPs*,*ZFP182* was involved in ABA-induced antioxidant defense and its overexpression significantly enhanced salt, cold, and drought tolerance in rice [82, 83].

Since these transcription factors might trigger the expression of Cd detoxification genes and, thus, converge Cd stress signals, further investigation on them and their downstream targets involved in Cd response will aid to unveil ZFPs-mediated regulatory networks in rice under Cd stress.

Cd exposure somewhat perturbs the expression of genes in drought, high salinity, and low-temperature stress-signaling pathways; therefore, 9% of Cd-upregulated transcripts, including *SNAC1* and *ZFP252*, were commonly upregulated among the 4 stresses [27]. Interestingly, 60 of the 164 universal Cd-responsive DEGs were Cd-upregulated and also commonly upregulated by the 4 stresses mentioned above (Table 2, Additional file 11: Table S5, Additional file 6: Dataset 1); therefore, these subset of 60 universal Cd-upregulated DEGs can be considered general stress-responsive genes. These include *OsNCED4* for ABA synthesis, *OsOPR1* for JA synthesis, *OsJAZ12* and *OsJAZ13* in JA signaling, 2 *GSTs*, and 7 TFs genes (*SNAC1*, *OsBIERF3*, 1 *MYB*, and 4 *ZFPs*) (Table 2). Regarding that overexpression of stress-inducible TFs can increase plant tolerance to multiple abiotic stresses, so upregulation of these general stress-responsive TFs could be useful for genetic improvement of crop tolerance to various abiotic stresses including diverse Cd pressure conditions.

### Cd stress-specific responsive genes

Except the 60 general stress-responsive genes, 104 universal Cd-regulated were not general stress-responsive and 14 of them were not reported to be Cd-responsive previously (Table 2; Additional file 11: Table S5). Among these 14 novel Cd-responsive DEGs, *Os04g33390* is the ortholog of Arabidopsis arogenate dehydratase 3 (*ADT3*). In plants, the arogenate pathway mediated by ADT isoforms is predominant in Phe biosynthesis [84], and *ADT3* supply of Phe is required to control reactive oxygen species (ROS) concentration and distribution to protect cellular components [85]. These indicate the potential role of this rice ADT3 ortholog in buffering and restricting ROS under Cd pressure.

Regarding the 104 Cd-specific regulated genes, several DEGs could be categorized into orthologous groups of model plants [86] (Table 2; Additional file 11: Table S5).

Of the 49 rice *OsPMEIs*, *OsPMEI46* and *OsPMEI28* are in the same orthologous group APK_ORTHOMCL14694 [87], and these two *PMEIs* are all upregulated by Cd treatment, though *OsPMEI28* (Os08g01670) is only in the list of short term High Cd-upregulated DEGs (Additional file 2: Table S2).

The activity of PME, responsible for catalyzing the demethylation of pectin in cell walls, is positively related to the binding of Cd and Al in plants [88]. Overexpression of one PME inhibitor (*OsPMEI28*) in rice resulted in dwarf phenotypes, and the cell wall thickness of culms of transgenic rice was greatly decreased [87]. Therefore, the roles of the two Cd-upregulated rice *PMEIs* in Cd stress tolerance and/or response need further investigation, especially the universal Cd-upregulated *OsPMEI46* should gain more attention.

Plant MMPs (matrix metalloproteases) have been implicated in programmed cell death, and in response to biotic and abiotic stresses [89–91]. Tomato Sl2- and Sl3-MMP act upstream of P69B (an extracellular protease of the subtilase family) in an extracellular proteolytic cascade that contributes to the regulation of cell death [90]. In 4-week-old Arabidopsis plants, *At2-MMP* was induced in leaves by Cd or methyl JA and in roots by NaCl; however, Cd inhibited its expression in inflorescence and leaves of 10-week-old plants [90, 92].

As *At2-MMP*, the two rice *MMPs* in group APK_ORTHOMCL1642 are not general stress-responsive, but they are universal Cd-upregulated genes in rice roots (Table 2). Therefore, the identification of the substrates of these proteases will provide further insight into the mechanisms of cell death control under heavy metal stress.

Phytocyanins (PCs) are ancient blue copper binding proteins function as electron transporter, and plastocyanins as well as uclacyanins are typical family members of PCs [93, 94]. Moreover, *PCs* may also function in enhancing resistance to stress [93]. Overexpression of an Arabidopsis blue copper binding gene (*AtBCB*) could confer Al resistance in Arabidopsis [95]. Transgenic tobacco lines overexpressing an early nodulin-like protein gene from *Boea crassifolia* (BcBCP1) showed enhanced tolerance to osmotic stress [96]. Amplification of the *PC* genes in monocot plants (e.g. maize and rice) was suggested to be a mechanism of tolerance to harmful stresses [93]. Here, 3 members of the plastocyanin-type PCs in the orthologous group APK_ORTHOMCL3089 are universal Cd-upregulated genes (Table 2). Furthermore, the other member within this group (*Os08g04340*) is also upregulated by high-Cd stress (Additional file 2: Table S2). So, addressing whether these Cd-upregulated rice *PCs* play key roles in Cd tolerance is valuable.

## Conclusion

To obtain more information from the RNAseq data on Cd-treated rice roots, the WGCNA package was employed to dissect the Cd-coupled co-expression gene modules from the filtered 22,080 transcripts across 17 RNAseq samples, including data from several published studies. Combined with the differential expression information, 164 universal Cd-responsive DEGs were identified as functioning under different concentrations of Cd across diverse time points.

More importantly, 104 of the 164 DEGs, including *ABCG36/OsPDR9* and *OsNAC3*, might specifically respond to various Cd pressures, and 14 members of these are also novel DEGs, including *OsJAZ9*, *OsNPR4* and *Os04g33390* (encoding prephenate dehydratase), only identified in this study through integrative analysis. Few studies have been performed on these Cd-specific responsive DEGs; because they are promising novel candidate genes, more systemic investigation on them is needed to gain a better understanding of plant responses to Cd stress.

## Methods

### Rice seedlings cultivation, Cd treatment, and samples preparation

The seedlings of rice (*Oryza sativa* spp. japonica cv. Nipponbare) were cultivated in a hydroponic system (Kimura B nutrient solution) [97] in a growth chamber with a temperature of 28/22 °C (day/night), photosynthetic active radiation of 200 μmol m^−2^ s^−1^, and a photoperiod of 14/10 h (day/night). All hydroponic solutions were continuously aerated and renewed every 3 days. When the third leaves were fully expanded, the seedlings were transferred into fresh growing solutions containing 100 μM CdCl_2_ for 1 h. Following Cd treatment, roots were collected from rice seedlings of uniform size and separated into three groups: untreated 0 h, untreated 1 h, and Cd treated 1 h (labeled as ck0h, CK1h and Cd1h, respectively). All experiments were performed at least twice with 3 biological replicates each, and 3 replication samples for each treatment were mixed into one for RNAseq analysis, as described previously [1, 2, 27].

### Rice roots RNA isolation, RNAseq library preparation and sequencing

Total RNA for RNAseq was extracted from rice seedling roots using a plant RNA kit (Omega, USA), and total RNA samples were then sent to Novogene Corporation (https://en.novogene.com/) for sequencing. Sequencing libraries were generated according to the manufacturer’s instructions (Illumina). Then the libraries were sequenced on an Illumina Hiseq 2000 platform and 100 bp paired-end reads were generated.

The process including RNA isolation, RNAseq library preparation and sequencing was repeated twice as 2 biological replicates. After the adaptor and low-quality sequences of pair-end reads were trimmed, a total of 32.63 Gb clean data from 6 cDNA libraries (labeled as ck0h, CK1h and Cd1h, each with 2 replicates) were obtained. Over 86% of the clean reads had scores at the Q30 level (Additional file 1: Table S1).

### Mapping pair-end reads to the rice reference genome

A comprehensive analysis pipeline was formulated and our workflow for analysis of RNAseq data was illustrated in Fig. 1. In brief, two ‘Tuxedo’ packages, TopHat-Cufflinks [98] and HISAT-StringTie-Ballgown [99], were utilized in parallel to process the RNAseq data and output per kilobase transcript per million reads mapped (FPKM) matrix for differential expression analysis (Fig. 1). The rice reference genome and gene model annotation files (MSU6) were downloaded from Illumina’s iGenomes project (support.illumina.com/sequencing/sequencing_software/igenome.html) directly. For the 6 rice roots samples (ck0h, CK1h and Cd1h, each with replicates) prepared in our lab, the clean reads per sample were aligned to the rice reference genome (Nipponbare) using TopHat2 (v2.0.11). For each sample, the mapped reads were then assembled into transcripts with Cufflinks package (v2.2.1). Index of the reference genome was built using Bowtie (v2.2.2) and paired-end clean reads were aligned to the reference genome using TopHat2. After alignment, Cufflinks was employed to assemble the transcripts. Gene- and transcript-level expression values were represented by FPKM and were measured with Cufflinks.

Then the Cuffdiff was performed to evaluate the differential expression of genes and transcripts across various conditions (Fig. 1). A stringent criteria (fold-change ≥ 2 and q_value ≤ 0.05) was used to screen DEGs between each set of compared samples. MapMan (v3.6.0 RC1) was then used to annotate and subsequently visualize the DEGs on metabolic pathways [100].

### Preprocessing public transcriptomic datasets of Cd-stressed rice roots

The RNAseq data (SRP053169, prepared in our lab) representing early transcriptomic response to high-Cd exposure (100 μM) were re-analyzed in combination with those published RNAseq data of Cd-treated rice seedling roots (DRP001141 and SRP058434, Additional file 1: Table S1), particularly from rice roots samples of medium Cd stress (50 μM) for 1 h and 24 h (designated MCd1 and MCdD, respectively) (single-end, each with 3 replicates, and only 0 h control ck0 available) [27], and high Cd pressure (100 μM) for 24 h (labeled as Cd24h) (pair-end, without replicate and the corresponding control ck24h available) [1]. In total, 17 datasets consisting of 3 time points and 3 concentrations of Cd were preprocessed by the ‘new Tuxedo’ RNAseq analysis package HISAT-StringTie-Ballgown (Fig. 1).

The RNAseq reads for each sample were mapped to the rice reference genome (MSU6) using HISAT2, and the output SAM files were sorted and converted to BAM files using SAMtools (version 0.1.19). Then the sorted alignments were assembled into transcripts and the expression levels of all genes and transcripts were estimated using StringTie. Similarly, StringTie merge function was performed after assembling each sample, then the merged transcripts are fed back to StringTie one more time so that it can re-estimate the transcript abundances using the merged structures, meanwhile create table counts for Ballgown [99].

Considering that some sample groups with less than three replicates or being single-end sequenced, the expression (FPKM values) matrix of all transcripts from Ballgown was extracted directly to analyze DEGs using the limma package [101]. The voom transformation was applied to the read counts. After this, the usual limma pipelines for differential expression was performed with the default stringent criteria (fold-change ≥ 2 and adj.P.Val ≤ 0.05).

### Co-expression network analysis of Cd-stressed roots transcriptomes with WGCNA package

To reveal potential Cd-responsive modules in the public temporal transcriptome data of roots under various Cd pressures, the WGCNA software package in R was used to construct gene co-expression network of Cd-stressed rice roots from the normalized log2-transformed FPKM matrix from the Ballgown output mentioned above. To be included in the WGCNA workflow, transcript isoforms needed to have no more than 50% missing data [40]. Based on these criteria, there were 22,080 transcripts filtered for WGCNA unsigned co-expression network analysis (Fig. 1).

WGCNA network construction and module detection was conducted using an unsigned type of topological overlap matrix (TOM) with a soft-thresholding power β of 7 (*R* ^2^ > 0.7), a minimal module size of 30 and a branch merge cut height of 0.25, as described in previous reports [30, 33, 37, 43, 102].

## Additional files

Additional file 1: Table S1. RNAseq information of rice roots under various Cd stresses conditions. (XLSX 12 kb)

Additional file 2: Table S2. Cd-responsive 1772 differentially expressed genes transcripts isoforms (DEGs) in rice roots exposed to Cd for 1 h. (XLSX 186 kb)

Additional file 3: Table S3. Primers for quantitative RT-PCR. (XLSX 9 kb)

Additional file 4: Figure S1. Quantitative RT-PCR of 8 randomly selected DEGs expression in in rice roots exposed to Cd for 1 h. *Actin-1* (LOC4333919) was used to standardize transcript levels in each sample. The primers used in the qRT-PCR experiments are listed in additional Table S3. (PPT 75 kb)

Additional file 5: Figure S2. A Venn diagram showing the intersections of Cd-responsive 1772 DEGs and those identified in previous two reports. Rice roots exposed to 1 h of Cd treatment (Cd1h) and its control (ck1h) are sampled in our lab. The published rice roots RNAseq data of medium Cd stress for 1 h (MCd1) [27], and the microarray analysis of low Cd stress for 3 h (lowCd) [21] are listed in Additional file 6: Dataset 1. (PPT 373 kb)

Additional file 6: Dataset 1. Rice roots short-term Cd-responsive DEGs identified in previous three reports. (XLSX 621 kb)

Additional file 7: Figure S3. Transport overview of 1772 DEGs in rice roots under Cd stress. DEGs were selected for the metabolic pathways analysis using the MapMan software (v3.6.0RC1). The colored boxes indicate the Log2 ratio of Cd1h/ck1h (1 h of Cd treated and untreated rice roots, respectively). (PPT 282 kb)

Additional file 8: Dataset 2. The FPKM matrix of 22,080 rice genes transcripts across 17 RNAseq samples. (TXT 2098 kb)

Additional file 9: Dataset 3. Four DEGs datasets (MCd1 vs ck0, MCdD vs ck0, Cd24h vs ck24h, Cd1h vs ck1h) output by limma package. (XLS 1903 kb)

Additional file 10: Table S4. One thousand eight hundred sixty-eight rice genes and transcripts in the Cd response-specific module generated by WGCNA. (XLSX 47 kb)

Additional file 11: Table S5. The expression of 164 universal Cd-responsive DEGs in Cd response-specific module across 4 DEGs datasets. (XLSX 38 kb)

## Abbreviations

ABC: ATP-binding cassette transporters
ADT: Arogenate dehydratase
ERF: Ethylene-response factor
GST: glutathione-S-transferase
HMA: Heavy metal ATPase
HMAD: Heavy metal associated domain containing protein
IRT1: Iron-regulated transporter 1
JA: Jasmonic acid
JAZ: JA ZIM-domain family protein
MATE: Multidrug and toxic compound extrusion
MMP: Matrix metalloendoproteinase
NCED: 9-cis-epoxycarotenoid dioxygenase
OPR: 12-oxo-phytodienoic acid reductase
PCs: Phytocyanins
PDR: Pleiotropic drug resistance
PMEI: Pectin methylesterase inhibitor
SNAC1: Stress-responsive NAC 1
WGCNA: Weighted gene co-expression network analysis
ZFP: Zinc finger protein
ZIPs: ZRT- and IRT-like proteins
