# Supplementary figures and images for: Co-expression network analysis of the transcriptomes of rice roots exposed to various cadmium stresses reveals universal cadmium-responsive genes
Source: BMC Plant Biol. 2017 Nov 7;17:194. doi: 10.1186/s12870-017-1143-y (PMC5678563; doi:10.1186/s12870-017-1143-y)

## Slide 1
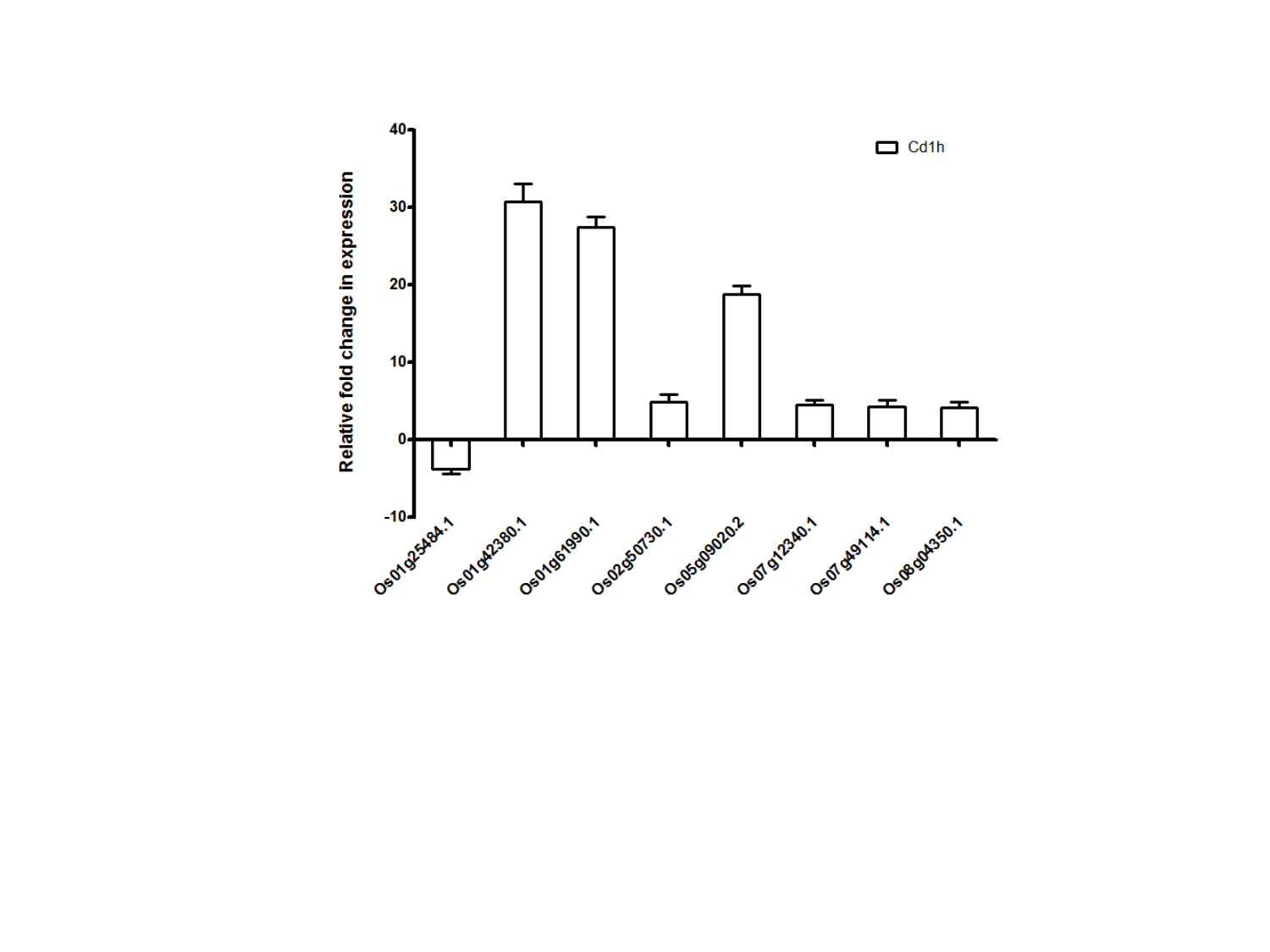

Supplement: Supplementary file 4 — Quantitative RT-PCR of 8 randomly selected DEGs expression in in rice roots exposed to Cd for 1 h. Actin-1 (LOC4333919) was used to standardize transcript levels in each sample. The primers used in the qRT-PCR experiments are listed in additional Table S3. (PPT 75 kb) [file 12870_2017_1143_MOESM4_ESM.ppt]

## Slide 1
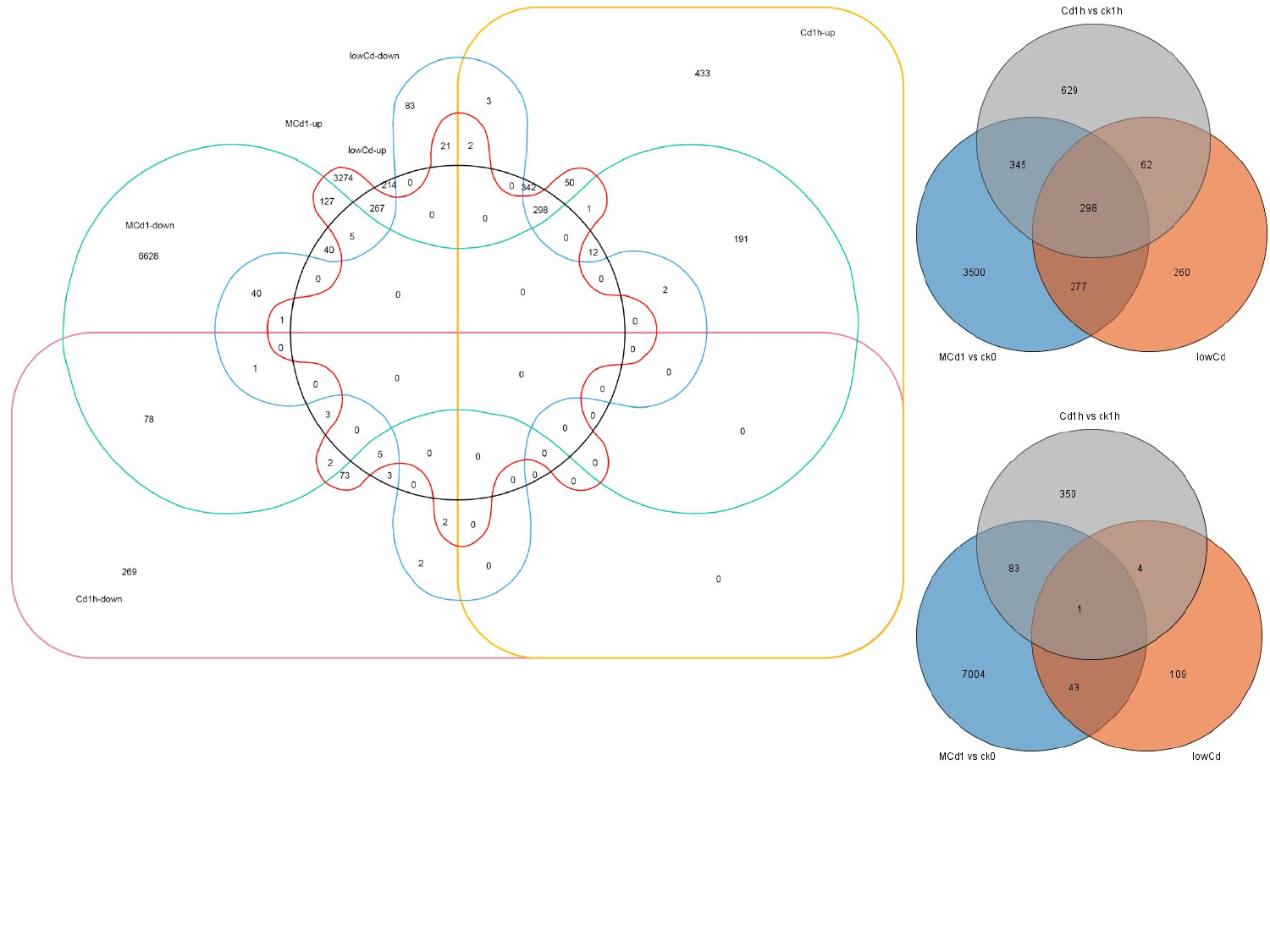

Supplement: Supplementary file 5 — A Venn diagram showing the intersections of Cd-responsive 1772 DEGs and those identified in previous two reports. Rice roots exposed to 1 h of Cd treatment (Cd1h) and its control (ck1h) are sampled in our lab. The published rice roots RNAseq data of medium Cd stress for 1 h (MCd1) [27], and the microarray analysis of low Cd stress for 3 h (lowCd) [21] are listed in Additional file 6: Dataset 1. (PPT 373 kb) [file 12870_2017_1143_MOESM5_ESM.ppt]

## Slide 1
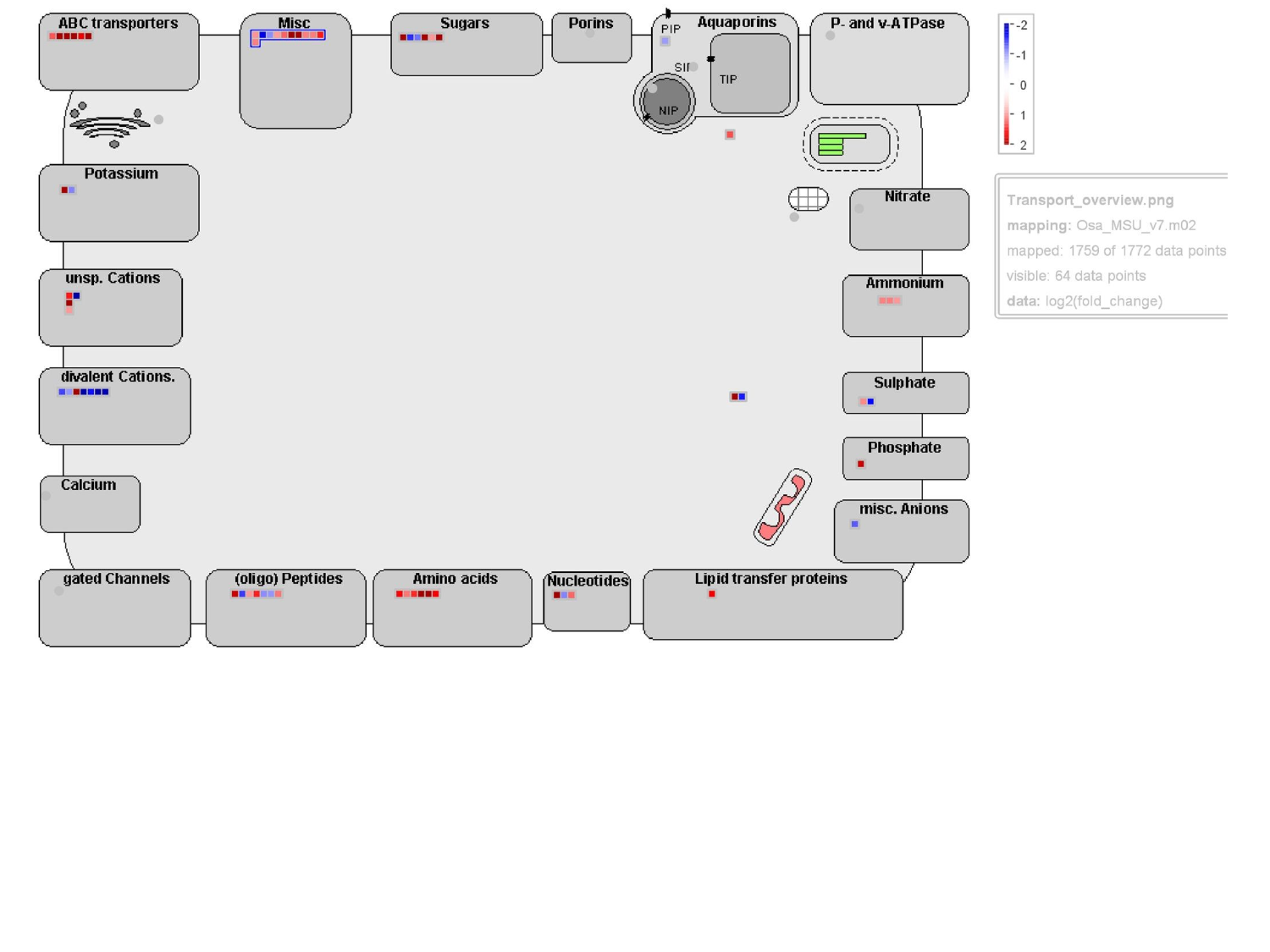

Supplement: Supplementary file 7 — Transport overview of 1772 DEGs in rice roots under Cd stress. DEGs were selected for the metabolic pathways analysis using the MapMan software (v3.6.0RC1). The colored boxes indicate the Log2 ratio of Cd1h/ck1h (1 h of Cd treated and untreated rice roots, respectively). (PPT 282 kb) [file 12870_2017_1143_MOESM7_ESM.ppt]
